# Risk factors for osteoporosis in liver cirrhosis patients measured by transient elastography

**DOI:** 10.1097/MD.0000000000010645

**Published:** 2018-05-18

**Authors:** Jian-ping Zheng, Hai-xiong Miao, Shao-wei Zheng, Wei-le Liu, Chu-qun Chen, Hao-bo Zhong, Sheng-fa Li, Yong-ping Fang, Chun-han Sun

**Affiliations:** a Department of Orthopedics; b Department of Hepatobiliary Surgery, Huizhou Medical Research Center, Huizhou First People's Hospital, Huizhou, Guangdong, China.

**Keywords:** body mass index, bone mineral density, fibroscan, liver cirrhosis, liver stiffness, osteoporosis

## Abstract

Osteoporosis or osteopenia is a common complication in patients with cirrhosis, but little is known about the risk factors for the occurrence of osteoporosis.

Patients with liver cirrhosis due to chronic virus infection and alcoholic abuse were enrolled. Bone mineral density (BMD) was determined using dual-energy x-ray absorptiometry (DXA). Osteoporosis was diagnosed according to WHO criteria. The severity of liver stiffness was measured by Fibroscan. Demographic data, such as age, gender, weight, height, and body mass index (BMI), were collected. Logistic regression analysis was used to recognize the risk factors of osteoporosis in patients with cirrhosis.

A total of 446 patients were included in this study: 217 had liver cirrhosis (male, 74.2%; mean age, 57.2 ± 10.27) and 229 were matched controls (male, 69%, mean age, 56.69 ± 9.37). Osteoporosis was found in 44 patients (44/217, 20.3%). The spine and hip BMD in cirrhotic patients were significantly lower than that in controls. When the cirrhotic and control subjects were stratified by age, gender, and BMI, the significant difference was also observed in women patients, patients older than 60, and patients with BMI < 18. Multivariate analysis showed that the older age [odds ratio (OR) = 1.78, *P* = .046], lower BMI (OR = 0.63, *P* = .049), greater fibroscan score (OR = 1.15, *P* = .009), and liver cirrhosis induced by alcohol liver disease (OR = 3.42, *P* < .001) were independently associated with osteoporosis in cirrhotic patients.

Osteoporosis occurred in about one-fifth of patients with liver cirrhosis, which was associated with age, BMI, Fibroscan score, and alcohol liver disease related liver cirrhosis.

## Abstract 2

Supplemental Digital Content is available in the text

## 1 Introduction

It was estimated that about 200 million people in the world suffered from osteoporosis, and its medical burden has been the 5th highest among the age-related diseases.^[1]^ Osteoporosis is characterized by decreased bone mass and strength, low bone mineral density (BMD), and eventually increased possibility for fractures.^[2]^ Both the prevalence and awareness of osteoporosis are increasing, as the aging populations increase worldwide.

In recent years, osteoporosis has been increasingly found in patients with chronic liver diseases, especially cholestatic liver disease.^[3]^ Furthermore, the prevalence of osteoporosis is reported as high as 55% of patients with advanced liver cirrhosis resulting from any causes.^[4–6]^ The huge diversity of prevalence maybe, at least in part, attributed to the ambiguous define of osteoporosis in patients with liver diseases. Hepatic osteodystrophy was created by many authors, which include osteopenia and osteoporosis associated with chronic liver disease.^[7]^ However, some studies did not differentiate the osteoporosis from hepatic osteodystrophy. On the contrary, the different reported prevalence of osteoporosis maybe attributed to the fact that liver cirrhosis can be caused by many factors, such as hepatitis B virus (HBV) infection, alcoholic abuse, and autoimmune hepatitis. However, patients with autoimmune diseases require glucocorticoid consumption, which is a main cause of secondary osteoporosis. Hence, it is necessary to characterize the feature of osteopenia and osteoporosis separately, with a diagnostic criterion and a defined population.

It has been suggested that the degree of liver cirrhosis, gender, and age were considered to be risk factors for the development of osteoporosis in patients with primary sclerosing cholangitis.^[8]^ Although osteoporosis or osteopenia is common in patients with cirrhosis, it remains unclear whether related risk factors, such as age, body mass index (BMI), and the severity of liver stiffness, can contribute to osteoporosis or osteopenia.^[9]^ The best strategy for the management of osteoporosis or osteopenia in patients with cirrhosis is primary prevention. Therefore, it is important to identify the risk factors of osteoporosis to alleviate the burden of osteoporotic fractures. In addition, there are conflicting results for the associations between the severity of liver cirrhosis and the osteoporosis in patients with cirrhosis.^[10–12]^

Therefore, the objectives of our study were to characterize osteoporosis or osteopenia in patients with cirrhosis; and to recognize risk factors of osteoporosis for patients with cirrhosis.

## 2 Methods

### 2.1 Subjects

The patients with liver cirrhosis caused by viral hepatitis or alcohol abuse, who were admitted in the Huizhou First Hospital between January 2015 and December 2016, were enrolled in the present study, irrespective of their baseline BMD. The liver function of all patients included in the study were Child–Pugh A. In addition, patients were excluded if they suffered from primary biliary cirrhosis (PBC), primary sclerosing cholangitis (PSC), and autoimmune hepatitis.^[13]^ All liver cirrhosis patients included in the current study underwent either liver biopsy or computed tomography and magnetic resonance imaging. The diagnosis of liver cirrhosis was based on liver histopathological examination, computed tomography, or magnetic resonance imaging. The patients without liver diseases, who matched with age and gender, were collected as controls. Exclusion criteria included younger than 18 years, glucocorticoid-induced osteoporosis, renal insufficiency, current use of medications for osteoporosis, metabolic bone disease, a recent history of major upper gastrointestinal disease, or a history of cancer other than hepatocellular carcinoma within the last 5 years. The demographic and clinical characteristics, including age, sex, weight, height, BMD, and fibroscan score, were extracted from clinical databases. None of the authors has access to get the identical information of subjects after data collection.

The Institutional Review Board of Huizhou First Hospital approved this study. All procedures were in accordance with the ethical standards of the responsible committee on human experimentation and with the Helsinki Declaration revised in 2008.

### 2.2 Dual-energy x-ray absorptiometry (DXA)

BMD was determined using dual-energy x-ray absorptiometry (DXA; Lunar PIXI; General Electric Healthcare, Madison, WI). The scans included a posteroanterior position and supine lateral position for the lumbar spine, and femoral neck for the hip. The scan procedure was performed as recommended by the manufacturer. The posteroanterior spine and hip scans were performed using the medium array mode, and the lateral scans using the fast array mode. The BMD was presented as measured values (g/cm^2^). According to the criteria of WHO, osteoporosis is defined by BMD at the hip or spine less than or equal to 2.5 standard deviations below the mean BMD of a young-adult reference population (*T*-score at or below  − 2.5), while osteopenia is defined by BMD between 1.0 and 2.5 SD below that of the mean level for a young-adult reference population (*T*-score between  − 1.0 and  − 2.5). In addition, hepatic osteodystrophy was defined as suffering from osteoporosis or osteopenia related to hepatic diseases.

### 2.3 Fibroscan examination

The severity of liver stiffness was measured by Fibroscan (vibration-controlled transient elastography; Echosens Corp., Paris, France) as described recently in detail by Zeng et al.^[14]^ Briefly, the tip of the transducer probe was placed on the upper abdominal skin over the right lobe of the liver. Measurement depth ranged from 25 to 65 mm beneath the skin surface. Ten measurements were carried out, and the IQR (interquartile range) did not exceed 40% in any of the measurements. The results were expressed in kilopascals (kPa). The median value was taken as representative.

### 2.4 Statistical analysis

Demographic data, including age, sex, weight, height, BMD, and Fibroscan score, were presented as mean ± SD. We assessed the possible risk factors of osteoporosis according to subgroup analyses (classified according to age, sex, BMI, and eteologies). The differences of demographic characteristics between the 2 groups were examined using *t* test. Multiple logistic regressions were used to determine the associations between osteoporosis and fibroscan score, height, weight, BMI, and biochemical findings, controlling for age and sex. Data management and statistical analyses were performed using SPSS for Windows, version 13.0 (SPSS Inc., Chicago, IL), with a *P* < .05 indicating statistical significance.

## 3 Results

### 3.1 Clinical data

**Figure 1:**
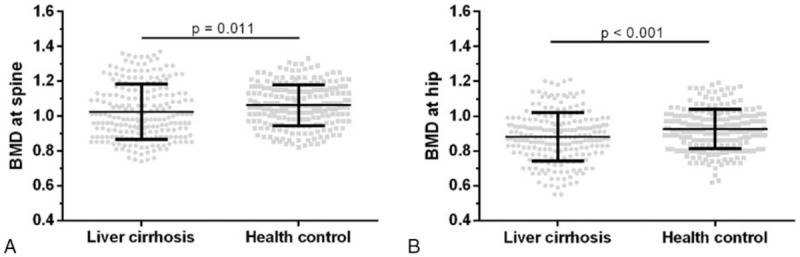
The spine and hip BMD in liver cirrhosis group and control group. As shown in Fig. 1, both spine BMD (A) and hip BMD (B) in patients with liver cirrhosis was significantly lower than that in controls. BMD = bone mineral density.

A total of 446 patients were enrolled in this study. Among them, 217 patients suffered from liver cirrhosis, and the other 229 subjects without liver diseases were considered as the control group. The characteristics of the subjects, including age, sex, height, weight, BMI, and fibroscan score, are presented in Table 1. There is no significant difference between the 2 groups for age, gender, and BMI. The height and weight of the cirrhotic subjects were lower than that of the controls (*P* < .05, Table 1). In addition, the Fibroscan score was significantly higher in the cirrhotic subjects than that of the controls. Spine BMD in the cirrhotics (1.02 ± 0.16) was significantly lower than that in controls (1.06 ± 0.16, *P* < .05, Fig. 1A). A similar trend can be observed in hip BMD (0.88 ± 0.14 vs 0.93 ± 0.11, *P* < .001, Fig. 1B).

**Table 1:**
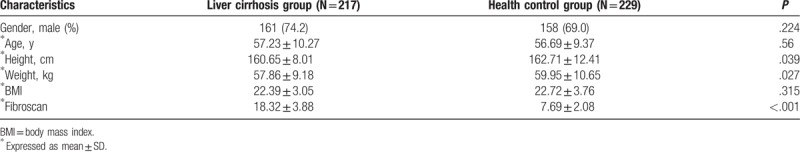
Characteristics of patients in liver cirrhosis group and control group.

### 3.2 The association between BMD and age, gender, and BMI

In general, greater age and less BMI were associated with lower spine and hip BMDs in both cirrhotic subjects and health controls. Men had greater BMD than women.

First, the cirrhotic and control groups were stratified by gender. The difference of hip and spine BMD between the cirrhotic and control subjects was significant in women, while the difference was present for hip BMD of men, but absent for spine BMD (Table 2).

**Table 2:**
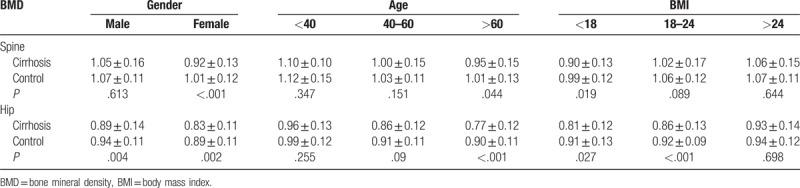
The subgroup analysis for the BMD according gender, age, and BMI.

Second, all the subjects were stratified into young (<40 years), mid-life (40–60 years), and old (age ≥ 60) subpopulations. For the old subpopulation, both the spine and hip BMD were significantly lower in patients with cirrhosis than that in controls (Table 2). However, for the young subpopulation, there is no significant difference in BMD between patients with cirrhosis and controls (Table 2).

Finally, the cirrhotic and control groups were stratified by BMI. For subjects having BMI < 18, both the spine and hip BMD was significantly lower in patients with cirrhotic than that in controls (both *P* < .05, Table 2). However, for subjects having BMI > 24, there is no significant difference in BMD between patient with cirrhosis and health controls.

### 3.3 The association between BMD and the etiologies of cirrhosis

**Figure 2:**
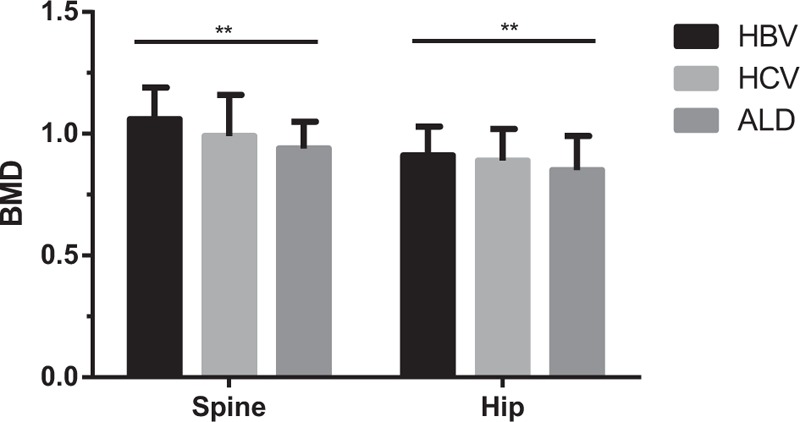
The difference of BMD in patients with liver cirrhosis caused by different etiologies. The mean BMD of patients affected by HBV, HCV, and alcohol abuse were 1.01 ± 0.18, 1.02 ± 0.18, and 0.94 ± 0.17 at spine and 0.85 ± 0.17, 0.87 ± 0.13, and 0.83 ± 0.12 at hip, respectively. There is a significant difference in spine and hip BMD among the 3 subgroups. The spine and hip BMD of patients affected by HBV were significantly greater than that of patients affected by alcohol abuse. ALD = alcoholic liver disease, BMD = bone mineral density, HBV = hepatitis B virus, HCV = hepatitis C virus. ^∗^*P* < .01; ^∗∗^*P* < .001.

To determine the association between osteoporosis or osteopenia and the etiologies of cirrhosis, we stratified the cirrhotics into 3 subgroups according to the etiologies, including HBV, hepatitis C virus (HCV), and alcohol abuse. There is significant difference in spine and hip BMD among the subgroups (Fig. 2). The spine and hip BMD of patients affected by HBV were significantly greater than that of patients affected by alcohol abuse (both *P* < .001), although there is no difference observed in HBV-related liver cirrhosis subgroup and HCV-related liver cirrhosis subgroup.

### 3.4 The characteristics of cirrhotic patients with osteoporosis and osteopenia

According to the diagnostic criteria of osteoporosis and osteopenia, 44 (20.3%) patients diagnosed as osteoporosis, 103 (47.5%) diagnosed as osteopenia, and 70 (32.2%) had normal BMD among patients with liver cirrhosis. The characteristics of the subjects are present in Table 3. Female patients were more likely to suffer from osteoporosis (*P* < .001). The patients with osteoporosis tended to have greater age (*P* < .001), greater fibroscan score (*P* < .05), and higher CRP level (*P* < .05).

**Table 3:**
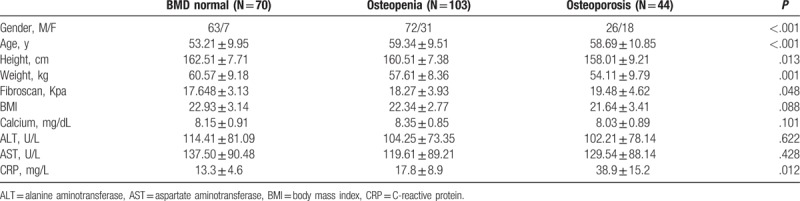
Baseline demographic and laboratory parameters of cirrhotic patients with normal BMD, osteopenia, and osteoporosis.

### 3.5 Risk factors for osteoporosis in patients with cirrhosis

Using logistic regression, the associations between osteoporosis and age, gender, BMI, fibroscan score, CRP level, and etiology of liver cirrhosis were analyzed, taking the patients with normal BMD and osteopenia as the reference (Table 4). Older patients tended to have osteoporosis [odds ratio (OR) = 1.78, *P* = .046]. Moreover, patients with liver cirrhosis are more likely to suffer from osteoporosis when they have lower BMI (OR = 0.63, *P* = .049) and more severe liver stiffness, indicating greater fibroscan score (OR = 1.15, *P* = .009). In addition, liver cirrhosis patients induced by ALD are more often to suffer from osteoporosis (OR 3.42, *P* < .001). We conducted logistic regression in the control group, as shown in supplementary Table 1. The result indicated that only female (OR = 3.97, *P* = .002) and older age (OR = 1.06, *P* = .001) were independent risk factors for osteoporosis in controls.

**Table 4:**
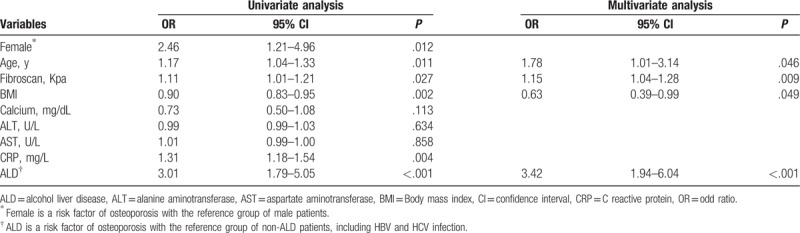
The association of demographic characteristics with osteoporosis in patients with liver cirrhosis: results from logistic regression.

## 4 Discussion

In this study, osteoporosis was found in a prevalence of 20.3% in patients with liver cirrhosis. In particular, patients with a higher fibroscan score and lower BMI do have a higher prevalence of osteoporosis.

The prevalence of osteoporosis was reported in 13% to 55% cases among patients with chronic hepatic diseases in western countries.^[15–17]^ Our study reported a prevalence of 20.3% in patients with liver cirrhosis caused by hepatic viral and alcoholic abuse. The wide variability of reported prevalence of osteoporosis maybe due to different etiologies and ages of the patients.^[6,18]^ In our study, we excluded patients with autoimmune liver cirrhosis because the osteoporosis maybe caused by glucocorticoid consumption, which is required for the treatment of autoimmune liver cirrhosis. Also, this is why that the prevalence rate is relatively lower than the previous studies.

Although there are a lot of reports involving the relationship between decreased bone mass and the Child–Pugh score, conflicting findings exist. As the decompensated liver cirrhosis patients had accompanied with a series of conditions, including elevated bilirubin and ascites. The symptoms such as ascites would affect the patients’ weight and BMD assessment. Hence, in this study, all included patients are compensated cirrhosis patients with liver function being Child–Pugh A.

Fibroscan is a noninvasive and quantitative technique to determine the liver stiffness severity. There is a rare study concerning relationship between the occurrence of osteoporosis and fibroscan score. Liver function plays an important role in regulating bone metabolism. Hypogonadism is an established risk factor for osteoporosis; thus, chronic hepatic diseases will accelerate the development of hypogonadism due to both reduced hypothalamic release of gonadotrophins and primary gonadal failure.^[19]^ In addition, a decline in level of estrogen in circulating may be another mediator of osteoporosis or osteopenia among patients with liver cirrhosis.^[20]^ Vitamin D3 is synthesized in the skin or absorbed through the gut, and then is hydroxylated in the liver by 25-alpha-hydroxylase and in the kidney by 1-alpha-hydroxylase, resulting in the formation of the active metabolite.^[21]^ Patients with cirrhosis often have vitamin D3 deficiency.^[22]^ Our study revealed that the severity of liver stiffness measured by Fibroscan significantly correlated with the occurrence of osteoporosis among cirrhotic patients. Therefore, we suggest that patients with a high Fibroscan score should have BMD monitoring regularly to have an early diagnosis and allow early intervention to avoid osteoporotic fractures.

It has been reported that age is one of the most important factors related to osteoporosis or osteopenia.^[23]^ Epidemiological surveys revealed that 10 million individuals older than 50 years have osteoporosis in the United States, and they have about 1.5 million osteoporotic fractures each year.^[24]^ Moreover, it was estimated that 27.6 million people in Europe had age-related osteoporosis and that more than 3.5 million fractures occur there each year.^[25]^ Many guidelines state that DXA screening should begin at 65 years for women. Because osteoporosis is usually asymptomatic until patient experience a hip or vertebral fracture. Therefore, osteoporosis can be disastrous for patients with liver diseases. Thus, early diagnosis is the key for appropriate osteoporosis management, allowing for adequate prevention and treatment in cirrhotic patients. Our study suggests that for cirrhotic patients, the age for initiating the DXA screening protocol should be earlier.

Weight loss is associated with a general increased risk of fractures,^[26]^ whereas weight gain is associated with a reduced risk of hip fractures.^[27]^ Low BMI was another risk factor associated with osteoporosis in patients with PBC.^[28]^ Consistently, our study implies that lower BMI was also a risk factor for osteoporosis in patients with noncholeastatic liver cirrhosis.

In accordance with previous findings, patients with alcoholic liver disease are more likely to develop osteoporosis or osteopenia, compared with patients with chronic viral hepatitis. This may be due to the fact that consumption of alcohol could cause decrease on the numbers and activity of osteoblasts, and impaired nutrition and hormone secretion for bone remolding.^[8,29]^

In addition, activation of inflammatory cells in patients with liver cirrhosis promote the production of pro-inflammatory factors such as tumor necrosis factor (TNF) and interleukin (IL)-1, which can decrease bone mass. Our study also demonstrated that the CRP level is significantly different between patients with normal BMD and patients with osteoporosis, indicating that inflammation may be involved in the pathogenesis of osteoporosis.

Some potential limitations of our study need to be discussed. The study sample size was relatively small, and therefore, the results may be biased. The data of this study were obtained from 1 center, which may introduce some interpretation bias. Thus, a multicenter study with larger sample sizes is needed. Although viral load is a risk factor for poor outcomes in viral hepatitis,^[30,31]^ we did not detect an effect of viral load on osteoporosis or osteopenia.

In conclusion, osteoporosis is commonly seen in patients with liver cirrhosis. Furthermore, patient with higher Fibroscan score and lower BMI are significantly associated with osteoporosis. Elderly cirrhotic patients are more likely to suffer from osteoporosis.

## Acknowledgment

We wish to thank Shawn for his helpful assistance with the manuscript, and for his rich knowledge of hepatology and skill in measuring liver stiffness by Fibroscan.

## Author contributions

Yong-ping Fang and Chun-han Sun designed the research; Jian-ping Zheng, Hai-xiong Miao, and Shao-wei Zheng performed the research; Wei-le Liu and Chu-qun Chen analyzed the data; Hao-bo Zhong, Sheng-fa Li, and Wei-le Liu wrote the paper.

**Conceptualization:** Yongping Fang, Haobo Zhong, Shengfa Li.

**Data curation:** Jianping Zheng, Haixiong Miao, Shaowei Zheng, Weile Liu, Yongping Fang, Haobo Zhong, Shengfa Li, Chunhan Sun.

**Formal analysis:** Shaowei Zheng, Weile Liu, Chuqun Chen, Haobo Zhong, Shengfa Li, Chunhan Sun.

**Funding acquisition:** Chunhan Sun.

**Investigation:** Jianping Zheng, Haixiong Miao, Chuqun Chen, Shengfa Li, Chunhan Sun.

**Methodology:** Yongping Fang, Shengfa Li, Chunhan Sun.

**Project administration:** Jianping Zheng, Chunhan Sun.

**Supervision:** Weile Liu, Chuqun Chen, Yongping Fang.

**Validation:** Chunhan Sun.

**Writing – original draft**: Jianping Zheng.

**Writing – review & editing:** Jianping Zheng, Chunhan Sun.

## Supplementary Material

Supplemental Digital Content
