# Supplementary material for: Risk factors for osteoporosis in liver cirrhosis patients measured by transient elastography
Source: Medicine (Baltimore). 2018 May 18;97(20):e10645. doi: 10.1097/MD.0000000000010645 (PMC5976349; doi:10.1097/MD.0000000000010645)
Supplement: Supplemental Digital Content [file medi-97-e10645-s001.docx]

| **Variables** | **Univariate analysis** | | |  | | **Multivariate analysis** | | | |  |
| --- | --- | --- | --- | --- | --- | --- | --- | --- | --- | --- |
|  | **OR** | **95% CI** | **P** | |  | | **OR** | **95% CI** | **P** | |
| ^a^Female | 4.50 | 1.92-10.56 | 0.001 | |  | | 3.97 | 1.67-9.45 | 0.002 | |
| Age, y | 1.06 | 1.03-1.09 | <0.001 | |  | | 1.06 | 1.02-1.09 | 0.001 | |
| BMI | 0.92 | 0.84-1.01 | 0.074 | |  | |  |  |  | |
| Calcium, mg/dl | 0.96 | 0.86-1.09 | 0.542 | |  | |  |  |  | |
| ALT, U/L | 0.99 | 0.99-1.01 | 0.424 | |  | |  |  |  | |
| AST, U/L | 0.99 | 0.99-1.02 | 0.311 | |  | |  |  |  | |
| CRP, mg/L | 0.83 | 0.91-1.12 | 0.827 | |  | |  |  |  | |

**Supplementary Table 1: The association of demographic characteristics with osteoporosis in control group**

ALT, alanine aminotransferase; AST, aspartate aminotransferase; BMI: Body mass index; CRP: C reactive protein; OR: odd ratio; CI: confidence interval.

^a^Female is risk factor of osteoporosis with the reference group of male patients.
